# Integration of auditory and tactile inputs to localize haptic stimuli during active touch

**DOI:** 10.3758/s13414-026-03223-w

**Published:** 2026-02-23

**Authors:** Giulia Esposito, Arthur S. Courtin, Olivier Collignon, André Mouraux

**Affiliations:** 1 Institute of Neuroscience, Université Catholique de Louvain, Avenue Mounier 53/B1.53.04, 1200 Brussels, Woluwe-Saint-Lambert Belgium; 2 Center for Functionally Integrative Neuroscience, Aarhus University, Aarhus, Denmark; 3 Institute of Research in Psychological Sciences, Université Catholique de Louvain, Louvain-la-Neuve, Belgium; 4 School of Health Sciences, HES-SO Valais-Wallis, The Sense Innovation and Research Center, Lausanne and Sion, Switzerland

**Keywords:** Active touch, Audio-tactile, Multisensory integration

## Abstract

Exploring our environment through touch often entails integration of tactile input with auditory and/or visual cues. The mechanisms by which mechanosensation integrates with other sensory modalities during active touch remain poorly understood, despite their ecological importance. Here, we investigated auditory-tactile integration in the context of edge localization during active tactile exploration. We assessed how accurately participants could determine the position of their moving finger in relation to the onsets of tactile, auditory, and auditory-tactile stimuli with respect to a visually displayed midline. We hypothesized that localization precision would be improved in the presence of combined auditory-tactile stimulation. In Experiment 1, the auditory, tactile, and auditory-tactile conditions were presented in separate blocks, while in Experiment 2, they were interleaved within blocks. For both experiments, we found that concurrent auditory-tactile stimulation did not increase localization precision. We also observed across all modalities an inclination to localize the finger position towards the right, possibly due to a shift induced by the left-to-right finger movement. This bias was reduced in the auditory-tactile condition of the second experiment, suggesting that when modality was not predictable, integration of auditory and tactile input may have led to a more accurate representation of finger position at stimulation onset. In conclusion, we show that combined auditory-tactile input may reduce biases in reconstructing the spatial location of a tactile stimulus generated by sliding the finger onto a flat surface. These observations have potential implications for the design of haptic technologies involving active touch.

**Supplementary Information:**

The online version contains supplementary material available at 10.3758/s13414-026-03223-w.

## Introduction

Touch is a dynamic process: We explore the world, its shapes, textures, and materials by actively interacting with them. While passive touch is defined as the experience of being touched, active touch is defined as an intentional act performed to optimally extract tactile information from the external world (Gibson 1962; Lederman and Klatzky 1993). Localizing stimuli in the external world through active dynamic touch requires not only the ability to localize where tactile stimuli occur on the body surface but also entails the ability to integrate this somatotopic information with information on where the touched body surface was located in external space at the onset of tactile stimulation. This process often engages the integration of signals from proprioception (e.g., information on the location and movement of the exploring fingers), motor commands (e.g., the efference copy), and vision or audition when it is present (e.g., viewing the exploring fingers manipulating an object, or hearing the sounds generated by finger contact with a texture) (Gibson 1962; Prescott et al. 2011; Simões-Franklin et al. 2011; Sciutti et al. 2010; Lederman 1979). As such, active touch can be thought of as a dynamic, multisensory experience. However, studies investigating the integration of touch with other sensory modalities in the context of active tactile exploration remain scarce, despite their ecological validity (Matusz et al. 2019).

In the context of the spatial localization of auditory and tactile stimuli, some evidence exists for cross-modal effects in passive touch conditions. A well-established cross-modal interaction phenomenon in the spatial domain is the ventriloquist illusion (Bertelson and Aschersleben 1998; Bertelson and Radeau 1981), where localization of an auditory stimulus is biased towards the location of a concurrently presented visual stimulus (Bertelson and Aschersleben 1998; Alais and Burr 2004). Evidence for ‘‘tactile ventriloquism’’ has also been reported, with previous studies reporting that a sound can influence the perceived spatial position of touch (Bruns et al. 2011; Bruns and Röder 2010; Caclin et al. 2002; Plöchl et al. 2016; Vercillo and Gori 2015).

Even though such studies conducted in conditions of passive touch provide valuable information on cross-modal effects between touch and audition, they are of limited relevance for active touch. Indeed, in a static passive touch experiment, localization of the tactile stimulus relies solely on our ability to determine *where* it occurs on the body surface (spatial somatotopic discrimination) and where that body part is located in the external space. In contrast, in conditions of active dynamic touch, where one is actively exploring the environment with the fingertip, spatial localization in the external space of an object contacted by the fingers requires integrating information on *when* the tactile stimulus generated by the finger–object contact occurs (temporal discrimination) and *where* the exploring fingers are located in space (integration with proprioceptive, motor control, and/or visual information on finger position). Furthermore, the mechanical energy dissipated during a transient fingertip-object interaction in active touch will often generate—in addition to a tactile stimulus—a temporally coinciding auditory stimulus (e.g., the sound generated by knocking fingers on a door). In the context of multisensory integration in the temporal domain, temporal ventriloquism effects have been described, in which auditory stimuli can bias the perceived temporal onset of visual stimuli (Fendrich and Corballis 2001; Morein-Zamir et al. 2003; Vroomen and De Gelder 2004). Such temporal effects of multisensory interactions are particularly relevant to active touch where, as mentioned above, spatial information about limb position needs to be integrated with temporal information on the onset of a stimulus.

To investigate auditory-tactile interactions in conditions of active touch, we designed a psychophysical experiment aiming at evaluating the contribution and potential integration of transient tactile and auditory feedback during active sliding of the finger against a surface. A key feature of multisensory integration is multisensory enhancement, where behavioral performance in bimodal versus unimodal conditions improves the ability to detect and/or make judgements about stimuli and shortens reaction times (Stein and Stanford 2008). For instance, in passive touch conditions, synchronously presented vibrotactile stimuli have often been shown to increase the detectability and perceived loudness of faint auditory tones (Gillmeister and Eimer 2007). These findings are compatible with modern theories of perception which posit that, rather than being a simple read-out of physical stimuli, perception results from inferential processes integrating sensory evidence and prior knowledge (Aggelopoulos 2015; Rohde et al. 2016). This integration is usually thought to be approximately Bayesian, meaning that the contribution of different sources of information (prior expectations, sensory evidence from various modalities, etc.) are weighted by their precision (inverse uncertainty) (Rohde et al. 2016; Ernst and Banks 2002). If two stimuli are presented at different spatial locations, or at different temporal onsets, the perceived location/onset would be a compromise between these locations/onsets, weighted by the relative precision of the modalities. If two stimuli are presented at the same location in space or close in time, precise localization should be easier when several sensory cues are available as compared to a single cue. In this study, our aims were to investigate how well participants can evaluate, during an active touch exploration task, the position of their moving finger relative to the onset of a tactile, auditory, or auditory-tactile stimulus. Specifically, we tested whether bimodal auditory-tactile stimulation improves localization precision compared with both unimodal tactile stimulation and unimodal auditory stimulation, which would be consistent with multisensory enhancement effects. It is important to note that for active tactile exploration, localization refers to the process of integrating temporal (given by the onset of the stimulation) and spatial (given by proprioceptive, motor, and visual information about finger position) cues to determine where the exploring finger was at the time the stimulation was received.

## Methods

### Participants

The study received ethical approval by the UCLouvain Ethics Committee. Forty-five healthy participants (with no self-reported history of motor or auditory disorders, or loss of sensitivity at their hands) were recruited. Twenty participants were recruited for Experiment 1 (17 women, three men, ages 18–34 years), and 25 were recruited for Experiment 2 (18 women, seven men, ages 18–35 years). Data collection had to be discontinued for three participants who were recruited for Experiment 2 because they struggled to perceive the haptic stimuli. Additionally, two participants who were recruited for Experiment 2 struggled to properly identify the stimulus types during the experiment, which led to many stimulus repetitions. For this reason, the data from these participants were excluded from the final dataset. Thus, the final sample for Experiment 2 included 20 participants. All participants gave written informed consent prior to participation. The choice of sample size was based on previous studies which used the psi method to assess differences between experimental conditions in within-subject designs (Filbrich et al. 2017; Courtin et al. 2023).

### Experimental setup

**Fig. 1:**
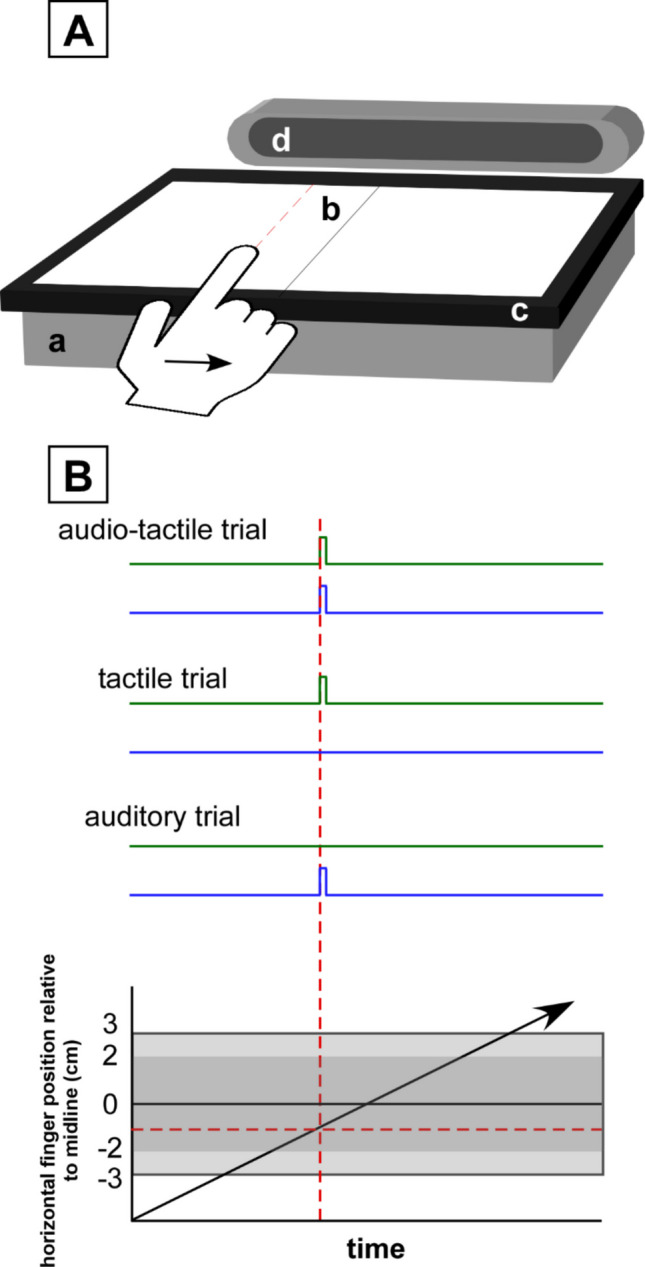
Experimental setup. **A** Participants were asked to slide from left-to-right their right index fingertip against a flat visuo-haptic display which renders tactile stimuli via friction-modulation (a). An infrared frame was placed on top of the display to monitor finger position and to trigger the auditory feedback (c), which was delivered via an external speaker placed just behind the haptic display (d). Participants had to report, after each trial, whether they perceived the stimulation (tactile, auditory, or audio-tactile) while their finger was located to the left or to the right of a visually displayed midline (b). The figure shows an example of a stimulus delivered while the fingertip was located to the left of the visually displayed midline (red dashed line). **B** Audio-tactile, tactile, and auditory stimuli were presented as follows. Participants performed horizontal left-to-right movements over the haptic display (bottom panel, diagonal arrow). Auditory (blue) and/or tactile (green) stimuli were triggered when the fingertip reached a given location on the haptic display (horizontal and vertical dashed red lines), relative to the visually displayed midline. The grey areas shows the range of locations that were used to estimate the psychometric curves in the two experiments. In Experiment 1 (darker grey area) the range was −2 cm to +2 cm. In Experiment 2 (lighter grey area) the range was −3 cm to +3 cm. (Color figure online)

Tactile stimuli were delivered using the E-VITA haptic display (Rekik et al. 2017) (Fig. 1A). This device works by means of amplitude-modulated ultrasonic vibrations, which render tactile feedback upon dynamic touch by reducing the coefficient of friction between the finger and the glass plate of the display. By controlling the vibration amplitude as a function of fingertip position, monitored using an embedded capacitive touch screen (Banana-LCD-5’’-TS, Marel, China, 50 Hz sampling frequency), the display can create tactile shapes or textures that are felt at the exploring fingertip at defined locations as it slides on the display (Rekik et al. 2017). The effect of ultrasonic vibration of the display on friction has not been completely elucidated, but might be mediated at least in part by ultrasonic lubrication and the ‘‘squeeze film’’ effect, where a thin layer of compressed air is created by the ultrasonic vibrations at the finger–surface interface (Wiertlewski et al. 2016).

An external infrared (IR) touch screen (Model TOT104UIR009, active area dimensions: 213 × 161 mm) frame was used to detect finger position (position accuracy: 2 mm) and thereby trigger the auditory stimuli at a given finger location on the tactile display (Fig. 1A).

### Tactile, auditory, and auditory-tactile sensory cues

The tactile (T) stimulus was a transient reduction of friction rendered by ultrasonic stimulation when the fingertip was located within an active area shaped as a vertical rectangle with a height of 100 mm and a width of 2 mm. In the context of planar shape exploration, a transient change in friction is expected to occur as the fingertip crosses the raised edges of the shape (Janko et al. 2018). The transient change in friction rendered via the haptic display used in this experiment thus artificially reproduced part of the tactile experience of crossing an indentation.

The auditory (A) stimuli were bursts of white noise lasting 22.7 ms delivered using the MATLAB Psychtoolbox. Transient white noise was selected because the sound elicited by the friction between a moving fingertip and a surface resembles white noise (Zahouani et al. 2009).

The bimodal auditory-tactile (AT) stimuli consisted of the same stimuli temporally synchronized and delivered when the fingertip reached a given position on the haptic display (Fig. 1B). Importantly, the auditory stimuli were delivered through a single small speaker placed just behind the tactile display, and were thus not spatially modulated according to when they were triggered depending on the location of the exploring fingertip (i.e., they were always delivered in the same way via the external speaker). Similarly, the tactile stimulus also remained unchanged relative to the fingertip’s location on the display (i.e., the same portion of fingertip skin was subjected to the transient change of friction). Localization of “where” the tactile, auditory and auditory-tactile stimuli occurred on the tactile display thus relied entirely on integrating temporal information about the onset of tactile and/or auditory feedback relative to spatial information about limb position provided by proprioceptive and/or visual feedback of fingertip location (Fig. 1B).

To avoid stimuli from one modality being perceived more strongly, a standard one-up, one-down staircase was used at the beginning of each experimental session, to match the perceptual intensities of the auditory and tactile stimuli. Participants were presented with AT stimuli and had to report which modality (tactile or auditory) was perceived as more intense. Since the intensity of tactile stimuli was kept constant across participants, only the volume of the auditory stimulus was adjusted with the staircase procedure. The staircase ended after ten reversals and the volume for the auditory stimuli was set to the average volume of the last nine reversals.

### Procedure

To allow participants to familiarize themselves with the stimuli and task, a brief training session was included. Participants were presented with stimuli placed at extreme distances from the midline (±3.5 cm, with steps of 0.5 cm, six per modality), for a total of 18 training trials, as described below.

#### Experiment 1 (blocked design)

Participants were asked to slide the index fingertip of their dominant hand in a horizontal left-to-right direction along the surface of the haptic display. To allow for more ecological tactile exploration conditions, participants were not constrained to a fixed scanning velocity. They were instructed to slide their finger simply avoiding extremely fast or extremely slow scanning speeds, which would have impaired perception of the tactile feedback delivered with the haptic display. On the display, a black vertical line (0.5 mm width) was displayed against a white background to mark the midline position. At the end of each fingertip slide, participants were asked to report whether they had perceived the tactile, auditory, or auditory-tactile stimulus while their finger was in the first half (left) or second half (right) of the display relative to the visual midline position (two-alternative forced-choice task; Fig. 1). Participants were informed about what modality they would receive (T, A, or AT) prior to each block, and were encouraged to report lack of or incorrect detection of the stimuli (e.g., only hearing the A stimulus in AT trials), in which case the trial was repeated.

The distance relative to the midline of the T, A or AT stimuli was determined on a trial-to-trial basis using the psi+ method, run using the Palamedes toolbox in MATLAB (Prins 2012; Prins and Kingdom 2018). The psi+ method is an extension of the original psi method proposed by Kontsevitch and Tyler (Kontsevich and Tyler 1999), a Bayesian adaptive algorithm aiming at optimizing stimulus placement to gain information on the psychometric function parameters: the threshold α (mid-point of the function), the slope β (rate of change parameter), and the lapse rate λ (determining the upper and lower asymptotes of the function). To do so, it keeps track of a joint probability distribution for the function parameters, selects stimulus placement to optimize entropy reduction on these parameters, updates the full joint distribution based on the response to the presented stimulus, and selects the next stimulus following the same approach for a given number of stimuli (Kontsevich and Tyler 1999).

The method was run separately for the different conditions (A, T, AT). The psychometric function shape to be estimated by the algorithm was set to a cumulative Gaussian. Given the nature of the task, classification errors at both extremes of the stimulus range were considered lapses and the upper and lower asymptote values of the psychometric function were estimated jointly. The method was constrained to select stimulus locations relative to the midline ranging from −2.0 cm to +2.0 cm in steps of 0.5 mm, and to consider threshold α values in the same range. It was also constrained to consider slope β values between 0.05 and 5, and lapse rate λ values between 0 and 0.2. Threshold (alpha) and slope (beta) priors were uniform on the predefined range. Lapse rate (lambda) prior was a half-normal with a mean of 0 and standard deviation of 0.5, normalized to integrate to 1 on the predefined range.

The experimental session comprised a total of 300 trials (100 per modality), divided into 12 blocks of 25 stimuli, four for each modality (T, A and AT). The order of the blocks was pseudo-randomized across participants: To avoid too many repetitions of blocks of the same modality, the order of the blocks was randomized for every sequence of three blocks (e.g., Block 1 = A, T, AT; Block 2 = T, AT, A).

#### Experiment 2 (interleaved design)

In Experiment 1, participants were informed about what modality would be presented to them prior to each block. Such a blocked design could have led participants to choose the sensory cue (tactile or auditory) that they perceived as more reliable during bimodal trials, thus disregarding the sensory cue from the other modality. For this reason, a follow-up experiment was conducted using an interleaved design mixing T, A and AT trials within each testing block. Participants were not informed about modality prior to each trial, and could therefore not choose to focus on one modality or the other during bimodal trials. The task performed by the participant was identical to Experiment 1. To ensure correct perception of each modality, participants were also asked, at the end of each trial, to report what type of stimulation they perceived (T, A or AT), in addition to reporting the perceived location of their finger at the time the stimulus was presented. In case of lack of detection or incorrect detection of a stimulus (e.g., only perceiving a T or A stimulus during AT trials), the trial was repeated, and participants were not informed that they would be presented with the same stimulus.

The same psi+ adaptive procedure was employed. The stimulation and prior alpha ranges were increased compared to Experiment 1 from ±2.0 cm to ±3.0 cm as the results from Experiment 1 showed that, for some participants, the psychometric curve failed to saturate within the ±2.0-cm range (Supplementary Figs. 1 and 2). The prior beta (slope) and lambda (lapse) distributions were the same as in Experiment 1.

The experimental session comprised a total of 300 trials. The experiment was divided into 10 blocks, each comprising 30 trials (10 of each modality). Within each block, the order of presentation of the three conditions was interleaved and pseudo-randomized for each participant: to avoid too many repetitions of the same modality, the randomization procedure was repeated every six trials, thus obtaining two repetitions of stimuli of each modality within six trials (e.g., A;T;A;AT;T;AT – T;AT;T;A;A;AT).

### Data analysis

Rather than using the estimates of the psychometric function’s parameters returned by the psi+ method (threshold, slope, and lapse rate), we decided to use the raw stimulus–response pairs to fit new hierarchical models able to better account for the structure of the data (condition within participants, participants within group). This was motivated by the fact that independent psi+ runs need to be used for each participant and condition, corresponding to untenable assumptions of total independence between participants and conditions, and by the fact that we would have to either drop the uncertainty of these estimates and treat them as single observations (rather than as the results of fitting a model to 100 trials) or use complicated meta-analysis techniques to weight these estimates by their uncertainty.

#### Model choice

Assuming sensory noise to be the sum of many independent sources, this noise should be approximately normally distributed in accordance with the central limit theorem (Kingdom and Prins 2010). For such theoretical reasons, we chose to use a probit (i.e., cumulative normal) regression model, which is regarded as *‘‘perhaps the most justifiable form’’* of psychometric function (Kingdom and Prins 2010).

In order to assess within participants differences in threshold and slope with the modelling, the AT condition was chosen as the intercept of the linear predictors, and the difference between AT and T and between AT and A were modelled. As stimuli located at more positive values along the *x*-axis of the display (i.e., more to the right) should lead to a larger probability of reporting a stimulus on the right half of the screen, the slope was constrained to be positive by making it a base 10 exponential. As we did not expect the different conditions to affect lapses, the lapse rate was treated as a nuisance parameter, and was allowed to vary between participants, but not between conditions.

This gives us the following model:

$$For\ i\ in\ 1\ to\ I$$

$$For\ k\ in\ 1\ to\ K$$

$${y}_{i} \sim Binomial \left({\varphi }_{i}, {n}_{i}\right)$$

$${\varphi }_{i} = {\lambda }_{k} + \frac{\left(1-2{\lambda }_{k}\right)}{2} . erfc\left(\frac{-{\beta }_{k} . \left({x}_{i}-{\alpha }_{k}\right)}{\sqrt{2}}\right)$$

$${\alpha }_{i}= coe{f}_{\alpha A{T}_{k\left[i\right]}}+ coe{f}_{\alpha AT-{T}_{k[i]}}. {isT}_{i} +coe{f}_{\alpha AT-{A}_{k[i]}} . {isA}_{i}$$

$${\beta }_{i}= {10}^{\wedge }\left(coe{f}_{\beta A{T}_{k\left[i\right]}}+coe{f}_{\beta AT-{T}_{k[i]}}. {isT}_{i}+coe{f}_{\beta AT-{A}_{k[i]}}. {isA}_{i}\right)$$

$${\lambda }_{i}=\ \frac{1}{1+{e}^{-coe{f}_{{\lambda }_{k\left[i\right]}}}}$$

$$coe{f}_{\alpha A{T}_{k}}\sim Normal\left({\mu }_{\alpha AT}, {\sigma }_{\alpha AT}\right)$$

$$coe{f}_{\alpha AT-{T}_{k}}\sim Normal\left({\mu }_{\alpha AT - T}, {\sigma }_{\alpha AT - T}\right)$$

$$coe{f}_{\alpha AT-{A}_{k}}\sim Normal\left({\mu }_{\alpha AT - A}, {\sigma }_{\alpha AT - A}\right)$$

$$coe{f}_{\beta A{T}_{k}}\sim Normal\left({\mu }_{\beta AT}, {\sigma }_{\beta AT}\right)$$

$$coe{f}_{\beta AT-{T}_{k}}\sim Normal\left({\mu }_{\beta AT - T}, {\sigma }_{\beta AT - T}\right)$$

$$coe{f}_{\beta AT-{A}_{k}}\sim Normal\left({\mu }_{\beta AT - A}, {\sigma }_{\beta AT - A}\right)$$

$$coe{f}_{{\lambda }_{k}}\sim Normal\left({\mu }_{\lambda }, {\sigma }_{\lambda }\right),$$

where *K* is the number of subject,* k* is a varying index corresponding to the subject identity, *I* is the number of data points, *i* is a varying index corresponding to the current data point, *n* is the number of stimuli presented at a given intensity, φ is the probability to report the stimulus on the right part of the visually displayed midline, λ is the lapse rate, α is the threshold, β is the slope, *erfc* is the complementary error function, *isA* and *isT* are binary variables taking 0 (false) or 1 (true) value depending on the type of stimulus being used* (A: isA* = 1, otherwise*, isA* = 0; *T: isT* = 1, otherwise, *isT =* 0).

#### Model fitting

The model was fitted in *Stan* (Stan Development Team 2016) using the *Cmdstanr* package (Gabry et al. 2023) within the *R* statistical program (R Core Team 2021) and the R Studio interface (Allaire 2012). To fit the model, four chains of 5,000 iterations (including a warm-up period of 2,500 iterations, which was discarded) were used, leading to a total of 10,000 draws from the posterior distribution.

**Experiment 1.** A total of 1,958 stimulus–response pairs, coming from 20 participants and three conditions, were used to fit the model. Priors on standard deviations (σ) were constrained to be positive. The hyperprior (prior on group level parameter) for the intercept of the threshold predictors (µ_αAT,_ σ_αAT_) was a normal distribution with a mean of 0 and a standard deviation of 1. The hyperpriors for the slopes of the threshold predictors (µ_αT,_ σ_αT_; µ_αA,_ σ_αA_) were normal distributions with a mean of 0 and a standard deviation of 0.5. The hyperprior for the intercept of the log10(slope) predictors (µ_βAT_, σ_βAT_) was a normal distribution with a mean of 1 and a standard deviation of 0.5. The hyperpriors for the log10(slope) predictors (µ_βT_, σ_βT_; µ_βA_, σ_βA_) were normal distributions with a mean of 0 and a standard deviation of 0.25. The prior for the group mean of the logit transformed lapse rate was a normal distribution with a mean of −4 and a standard deviation of 1.

**Experiment 2.** A total of 2,067 stimulus–response pairs, coming from 20 participants and three conditions, were used to fit the model. The hyperprior for the intercept of the threshold predictors (µ_αAT,_ σ_αAT_) was a normal distribution with mean of 0 and a standard deviation of 1. The hyperpriors for the threshold predictors (µ_αT,_ σ_αT_; µ_αA,_ σ_αA_) were normal distributions with a mean of 0 and a standard deviation of 0.5. The hyperprior for the intercept of the log10(slope) predictors (µ_βAT_, σ_βAT_) was a normal distribution with a mean of 1 and a standard deviation of 0.5. The hyperpriors for the log10(slope) predictors (µ_βT_, σ_βT_; µ_βA_, σ_βA_) were normal distributions with a mean of 0 and a standard deviation of 0.25. The prior lapse rate was a normal distribution with a mean of −4 and a standard deviation of 1.

#### Model diagnostics

Following model fitting, appropriate sampling of the posterior distributions was assessed using the *ShinyStan* (Stan Development Team 2017): absence of divergent transitions, not reaching maximum tree depth, good alignment of energy diagnostic plots, E-BFMI larger than 0.2, effective sample size larger than 10% of the total sample size, Monte Carlo standard error smaller than 10% of the posterior standard deviation, and *Rhat* equal to or smaller than 1.01. Individual participant data fits were inspected visually as a posterior predictive check (see Supplementary Figs. 1–4).

#### Differences between conditions

For both experiments, the presence of significant interindividual threshold or slope differences between conditions *(*AT vs. T; AT vs. A; T vs. A) were assessed based on the posterior probability distributions of µ_αT_, µ_αA_, and µ_αA_ − µ_αT_ (thresholds) and µ_βT_, µ_βA_, and µ_βA −_ µ_βT_ (slopes). These distributions correspond to the population means of differences in threshold and slope between AT and T, between AT and A, and between A and T, respectively. These parameters were used to estimate the posterior probability (P) of our different null hypotheses by comparing 10^5^ random draws from their posterior distributions with 0. When comparing threshold estimates across conditions, bilateral tests were used, as we made no predictions on the effects of modality on threshold values within participants. For estimation of interindividual differences in slope, we conducted unilateral tests when assessing differences between the two unimodal and the bimodal conditions, given our preregistered (Experiment 1: 10.17605/OSF.IO/Y4GBN; Experiment 2: 10.17605/OSF.IO/X69UZ) hypothesis that concurrent auditory-tactile stimulation would improve precision, and bilateral tests when comparing the two unimodal conditions, since we did not have a preregistered hypothesis on the slope difference between these two. We considered the null hypothesis to be rejected when its posterior probability was less than 0.05.

To better illustrate the psychometric function fitted from the posterior distribution of the model parameters, an expected function was constructed, for each condition. This was achieved by constructing 10^4^ functions using random draws from the posterior distribution of the parameters and taking, for each temporal delay, the 50^th^ percentile of the stimulus detection probability. To get a sense of the uncertainty around these values, the pointwise 2.5^th^ and 97.5^th^ percentiles (95% credible interval) were also plotted. These psychometric functions can be interpreted as the expected values for a new unobserved participant coming from the same population.

## Results

### Experiment 1 (blocked design)

The model diagnostics revealed appropriate sampling, and the model appeared to fit well to the individual participants’ raw data (see Supplementary Figs. 1 and 2).

**Fig. 2:**
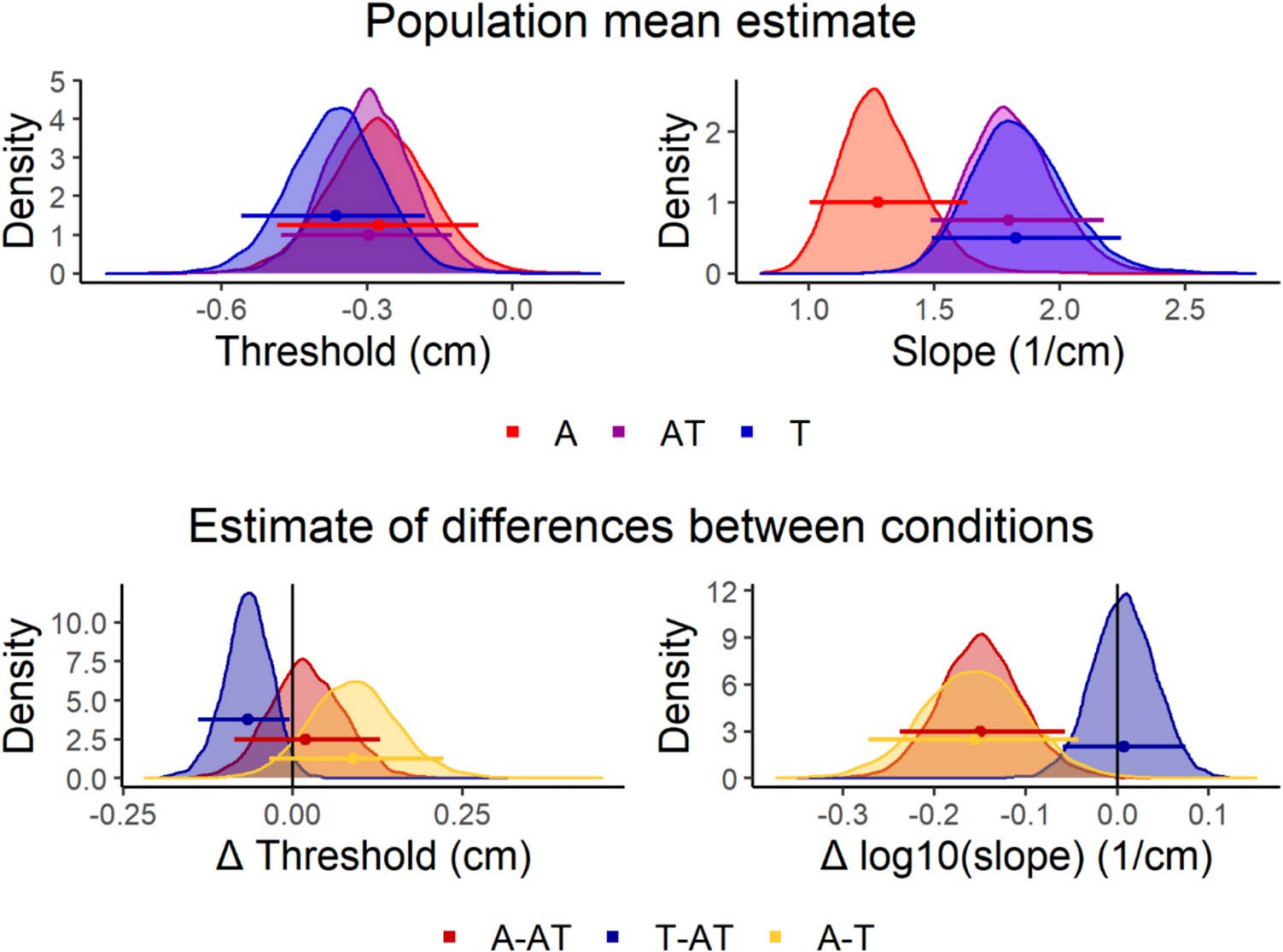
Posterior psychometric function parameter estimates for Experiment 1 (blocked design). The top row represents the population mean estimates for the threshold (left) and log10(slope) (right) for the three conditions. In the model, these parameters correspond to µ_αAT_/µ_βAT_ (AT), µ_αAT_ + µ_αT_/10^(µβAT + µβT)^ (T), and µ_αAT_ + µ_αA_/10^(µβAT + µβA)^ (A). The bottom row represents estimates of threshold (left) and log10(slope) (right) differences between conditions. In the model, these correspond to µ_αT_/µ_βT_ (T*-AT*) and µ_αA_/µ_βA_ (*A-AT*), and µ_αA −_ µ_αT_/µ_βA_ − µ_βT_ (*A*-T), where T-AT, A-AT, and A-T represent the population means of differences in threshold and slope between AT and T, between AT and A, and between A and T, respectively. In all cases, the horizontal lines represent the 95% highest probability density intervals of the posterior distributions and the dots represent the medians of the posterior distributions. (Color figure online)

As can be seen in Fig. 2, when the different conditions were presented in separate blocks (Experiment 1), thresholds of all three stimuli (A, T, AT) tended to be negative, indicating that participants reported that their finger was to the right of the midline when stimuli were presented close to it. Furthermore, the threshold for the tactile-only stimulus T was significantly more negative than that of the auditory-tactile stimulus AT (P_(T≠AT|data)_ = 0.034). The threshold of the auditory-only stimulus A appeared less negative than the thresholds of the other stimuli, but these differences were not statistically significant (P_(A≠AT|data)_ = 0.708, P_(T≠A|data)_ = 0.107). The slopes of the AT and T psychometric functions appeared very similar (P_(T<AT|data)_ = 0.581) and both were significantly greater than the slope of the A psychometric function _(_P_(A<AT|data)_ = 0.001, P_(T≠A|data)_ = 0.004), indicating greater localization precision in the case of tactile stimulation (T or AT).

**Fig. 3:**
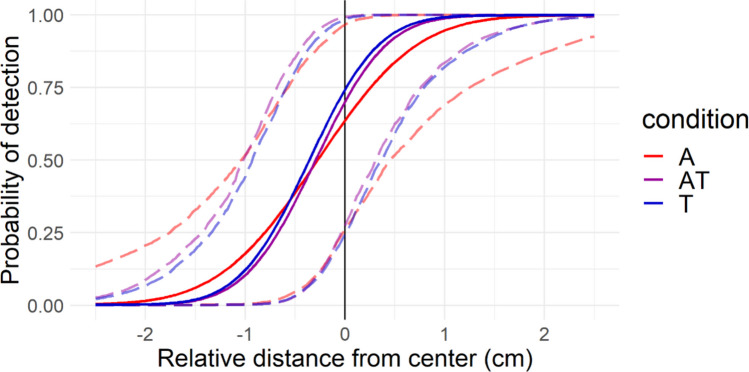
Posterior expected psychometric functions for Experiment 1 (blocked design). Bold lines represent the function constructed with the most likely parameter values (i.e., the median of 10^3^ random draws from the posterior distribution of the model parameters). Dotted lines represent the pointwise uncertainty around the posterior expected function (95% highest probability density intervals). (Color figure online)

A posterior predictive psychometric function, corresponding to what can be expected for a new participant coming from the same population taking Experiment 1, is displayed in Fig. 3.

## Experiment 2 (interleaved design)

The model diagnostics revealed appropriate sampling, and the model appeared to fit well to the individual participants’ raw data (see Supplementary Figs. 3 and 4).

**Fig. 4:**
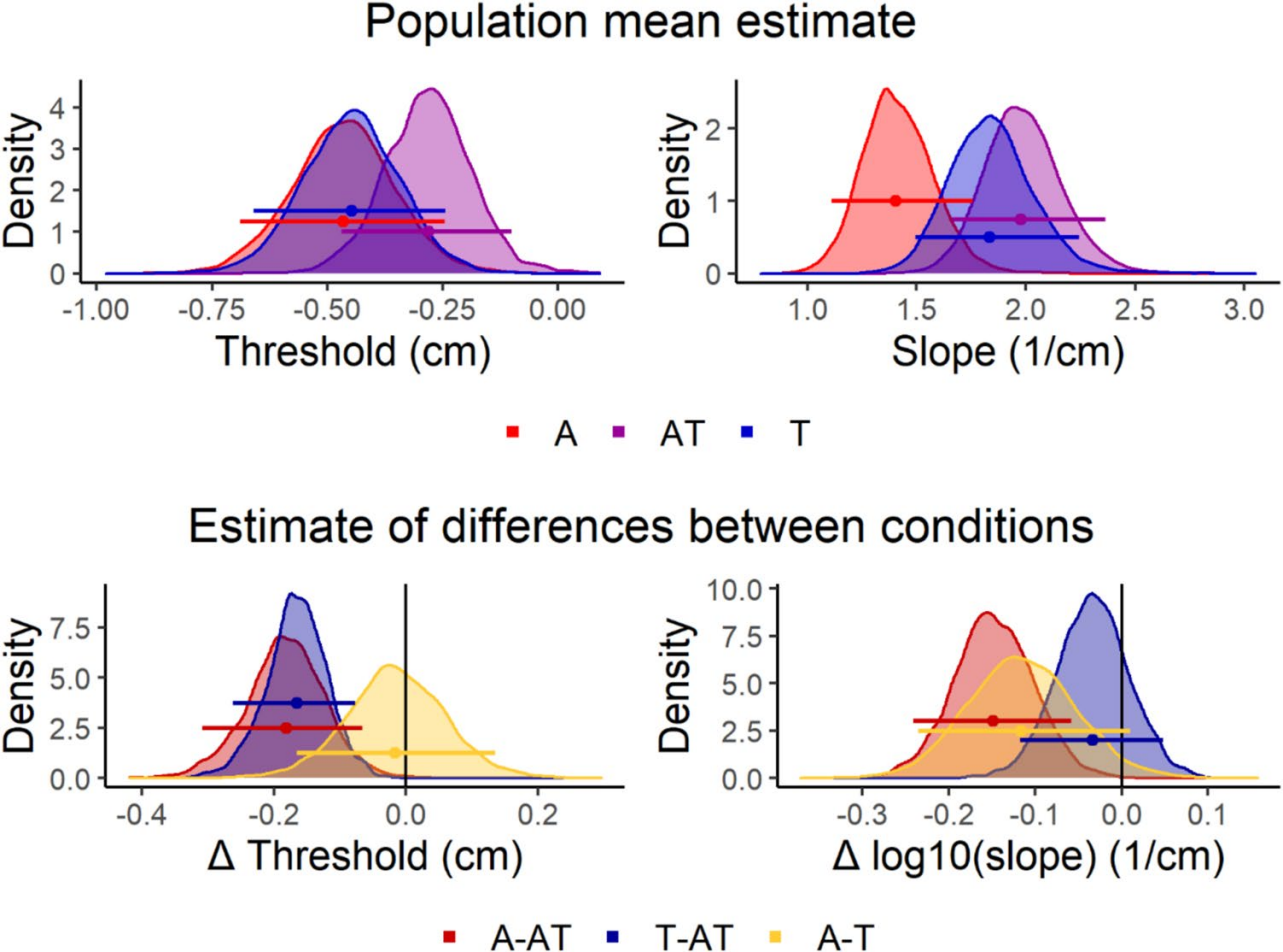
Posterior psychometric function parameter estimates for Experiment 2 (interleaved design). The top row represents the population mean estimates for the threshold (left) and log10(slope) (right) for the three conditions. In the model, these parameters correspond to µ_αAT_/µ_βAT_ (AT), µ_αAT_ + µ_αT_/10(^µβAT + µβT)^ (T), and µ_αAT_ + µ_αA_/10^(µβAT + µβA)^ (A),. The bottom row represents estimates of threshold (left) and log10(slope) (right) differences between conditions. In the model, these correspond to µ_αT_/µ_βT_ (T*-AT*) and µ_αA_/µ_βA_ (*A-AT*), and µ_αA −_ µ_αT_/µ_βA_ − µ_βT_ (*A*-T), where T-AT, A-AT, and A-T represent the population means of differences in threshold and slope between AT and T, between AT and A, and between A and T, respectively. In all cases, the horizontal lines represent the 95% highest probability density interval of the posterior distributions and the dots represent the medians of the posterior distributions. (Color figure online)

As can be seen in Fig. 4, when the different stimulus conditions were intermingled within the same blocks (Experiment 2), the threshold of the auditory-tactile AT stimulus was significantly less negative (closer to the display midline) compared to the auditory-only A stimulus (P_(T≠AT|data)_ = 0.005) and the tactile-only T stimulus (P_(A≠AT|data)_ < 0.001), which were extremely similar (P_(T≠A|data)_ = 0.801). The slope of the AT psychometric function appeared steeper than the slope of the T psychometric function (not significant; P_(T<AT|data)_ = 0.209) which itself was significantly steeper compared to the slope of the A psychometric function (P_(A<AT|data)_ = 0.001, P_(T≠A|data)_ = 0.028).

**Fig. 5:**
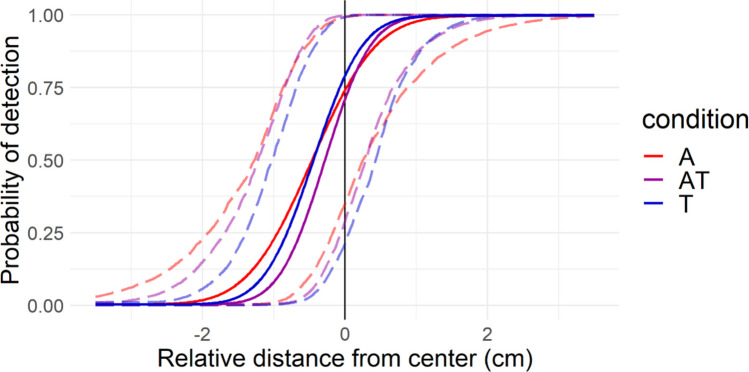
Posterior expected psychometric functions for Experiment 2 (interleaved design). Bold lines represent the psychometric function constructed with the most likely parameter values (i.e., the median of 10^3^ random draws from the posterior distribution of the model parameters). Dotted lines represent the uncertainty around the posterior expected function (95% highest probability density intervals). (Color figure online)

A posterior predictive psychometric function, corresponding to what can be expected for a new participant coming from the same population performing Experiment 2, is displayed in Fig. 5.

## Discussion

In this study, we aimed to investigate how observers integrate the onsets of auditory and/or tactile feedback to localize events in external space occurring during active tactile exploration (sliding the fingertip against a surface). Specifically, we assessed whether concurrent auditory-tactile stimulation improves localization performance compared with unimodal-tactile and unimodal-auditory stimulation. To address this question, two experiments were performed. In the first experiment, participants received information on the modality of the incoming sensory information prior to each block (auditory, tactile, or auditory-tactile). With such a design, participants could be inclined to ignore one of the sensory cues during bimodal trials, and simply respond to what they perceived as the most reliable cue. Therefore, in the second experiment, the three modalities were interleaved within blocks, and participants did not receive any information about the incoming sensory information prior to each trial. Results from both experiments revealed better localization precision (indexed by the slope of the fitted psychometric functions) for unimodal tactile stimulation compared with unimodal auditory stimulation. This suggests that the onset of tactile cues during fingertip sliding was more informative on localization than the onset of auditory cues. Given the nature of the task, where participants used their finger to actively explore a surface, it seems reasonable to expect a finer grained spatial representation of where the finger is when a tactile stimulus is perceived at its extremity than when a concurrent auditory stimulus without somatosensory counterpart is perceived.

While we had hypothesized that the combination of tactile and auditory sensory cues would lead to an improvement of localization precision, this was not the case in Experiment 1. In this experiment, participants knew in advance what type of stimulus would occur. It is thus likely that some participants focused on the more informative tactile modality when receiving bimodal stimuli, which poses a significant limitation to the interpretation of the group-level results from the first experiment. For this reason, the rest of the discussion will focus on the interpretation of the results from Experiment 2.

In that experiment, participants did not know in advance what type of stimulus they would receive and therefore could not beforehand focus on a specific modality. In this experiment, the slopes for the different psychometric functions followed our hypothesis (auditory < tactile < auditory-tactile), but the slope estimates between the T and AT modalities were not significantly different, whereas the slope estimate for the unimodal A condition was markedly lower than the other two conditions. This suggests that, such as in Experiment 1, tactile cues were more informative on localization as compared to auditory cues, and that combined auditory-tactile stimulation did not significantly improve localization precision.

The exploratory analysis of threshold estimates provides interesting insights in the effects of bimodal stimulus presentation on localization bias. In both experiments, and for all conditions, the psychometric curves were shifted towards negative threshold values. This indicates that, irrespective of modality, when stimuli were presented while the exploring finger was close to the midline, participants tended to respond that their finger was to the right of the midline. Several reasons could explain this effect. First, our results are consistent with leftward biases observed in bisection tasks (left side underestimation, or pseudo-neglect) frequently reported in the literature in healthy subjects, who tend to place their perceived midpoint towards the left of the true midpoint of a line (Bradshaw et al. 1985; Varnava et al. 2002). Second, the haptic display used to deliver the tactile stimuli modulates friction of the entire fingertip contacting surface, rather than a specific fingertip location. This is due to the fact that ultrasonic vibrations are triggered across the whole plate once the finger is detected within a predefined active zone (Vezzoli et al. 2016). Given that the display was explored in a single left-to-right direction, and taking into consideration the size of the fingertip contacting surface, it is therefore possible that the bias in the tactile modality was influenced by the fact that participants perceived the change in friction on the entire fingertip surface as soon as it entered the haptic rectangle. Thus, for stimuli presented at short distances to the left of the visual midline, the friction modulation also occurred at locations on the fingertip skin that had already crossed the midline, biasing perception to the right. Lastly, the observed perceptual rightward bias may relate to the effect of *representational momentum,* or forward displacement. This phenomenon occurs when, in the presence of motion, the localization of a stimulus is shifted towards the direction of movement (Freyd and Finke 1984; Hubbard 2005; Hubbard and Hubbard 2018; Merz et al. 2020). More specifically, our findings appear to be consistent with a special case of representational momentum, the flash-lag effect, which is observed when the position of a stationary object is assessed relative to a moving target object (here the position of the finger sliding against the display) (Hubbard 013). Different accounts of the mechanisms underlying the flash-lag effect have been proposed, including a longer processing time for stationary than for moving stimuli (Whitney et al. 2000), and the effect has already been described for haptic stimuli (the buzz-lag effect) (Cellini et al. 2016; Drewing et al. 2022; Drewing et al. 2018), and for cross-modal stimuli (Alais and Burr 2003). In our experiments, participants had to continuously integrate motor, proprioceptive, and visual inputs, to estimate the timing of stimulation relative to the estimated fingertip position, as neither the tactile nor auditory stimuli (corresponding to the flash in the flash-lag effect or the buzz in the buzz-lag effect) carried explicit spatial information in themselves (i.e., the somatosensory and auditory stimuli were identical regardless of where the finger was located on the haptic display). Since the task only involved left-to-right movements, it seems plausible that the estimated position of the exploring fingertip at the moment of transient auditory and/or tactile stimulation was shifted forward relative to the visually displayed midline, giving rise to a rightward perceptual bias in both experiments. In Experiment 2, where modality of the upcoming sensory cue was not predictable, bimodal stimulation led to a reduced bias compared with the unimodal auditory stimulation, suggesting that concurrent presentation of the auditory and tactile stimuli increased the temporal binding between the auditory-tactile stimuli and the estimated position of the exploring fingertip relative to the visually displayed midline. Such cross-modal effects on temporal binding have also been previously reported in the context of the flash-lag effect between the visual and auditory modalities (Vroomen and De Gelder 2004). In these experiments, it was shown that a sound concurrently presented with a visual flash significantly reduced the magnitude of the flash-lag effect, and that presenting the sound with an offset relative to the visual flash biased the effect linearly with the introduced delay, consistent with a temporal ventriloquism effect (Vroomen and De Gelder 2004). In drawing parallels between auditory-visual and auditory tactile interactions in the context of representational momentum, a key difference is that, while in the flash-lag effect the moving target is passively presented to participants (i.e., a dot moving horizontally across a screen), during the buzz-lag effect as well as in our task, the moving target is voluntarily displaced by the participants (i.e., the exploring fingertip). Localization during active finger sliding movements therefore relies on dynamically integrating spatial information about limb position at a given time (given by visual and/or proprioceptive cues) and temporal information about the onset of the tactile and/or auditory stimuli. In contrast with our results, a study investigating the effects of modality on the buzz-lag and flash lag-effects across vision and touch found that the magnitude of the effect was not different between unimodal versus bimodal conditions (Drewing et al. 2018). However, a key difference with the present work is that we tested differences between a bimodal (temporally synchronized auditory and tactile stimuli) and unimodal (tactile- or auditory-only) stimuli, while both the stationary object (the visually displayed midline) and the moving object (the exploring finger) were always the same across our experimental conditions. In line with our results, Drewing and colleagues (Drewing et al. 2018) reported that the characteristics of the flash (e.g., its intensity or duration) modulate the magnitude of the illusion, as they represent a temporal trigger to estimate the moving object’s position, and the time necessary to trigger the estimation depends on the processing timing of the flashed object (Drewing et al. 2018; Hubbard 2014). This is consistent with a redundant signals effect, which predicts that multisensory gain is reflected by faster response times in bimodal compared to unimodal conditions (Otto and Mamassian 2017; Raab 1962), and which has been reported in a large number of studies investigating audio-visual, visual-tactile and auditory-tactile interactions (Forster et al. 2002; Diederich and Colonius 2004; Zampini et al. 2007; Murray et al. 2001; Girard et al. 2013). In line with such gain effects, it is possible that, in the bimodal condition of our study, participants made quicker decisions about the stimuli, and therefore were faster at reconstructing the position of their finger relative to when the stimulus was perceived, thus leading to a more accurate localization of finger position relative to the stimulation onset. However, since we have no measure of reaction times to the stimulations used in our experiment, it is not possible to evaluate whether the effects we observed on accuracy could result from simple statistical facilitation and be driven by the fastest modality (as predicted by the race model (Otto and Mamassian 2017; Raab 1962)), or whether sensory integration took place prior to the decisional process (as predicted by the co-activation model (Otto and Mamassian 2017; Miller 1982). Furthermore, bimodal stimulation did not lead to significant improvements in precision (i.e., the slope of the psychometric function), possibly highlighting the fact that tactile stimuli provided sufficient information in isolation for participants to reach ceiling performance levels.

Importantly, the movement-related bias we observed could be intrinsic to different types of active tactile exploration. While the use of only one direction of movement does not allow us to fully exclude effects of left-side underestimation, previous studies that have reported buzz-lag effects in active touch employing both directions of movement have shown that the lagging effect occurs in both directions (Cellini et al. 2016). Therefore, it seems plausible to suggest that the bias we observed is more closely related to representational momentum. Our finding that concurrent sound reduces such bias could therefore have important implications for the design of haptic technologies.

In conclusion, we show that, under our experimental conditions, concurrent auditory stimulation did not lead to a significant improvement of the localization precision of the fingertip at the time it receives a tactile stimulus in conditions of active touch, possibly due to the higher reliability and relevance of fingertip tactile input compared to auditory input during tactile exploration. Interestingly, however, our exploratory analysis revealed a localization bias compatible with the audiovisual flash-lag effect or the tactile-visual buzz-lag effect, which was significantly reduced under bimodal stimulus presentation. Therefore, cross-modal effects between sound and touch in conditions of active haptic exploration could increase the temporal binding between transient auditory-tactile cues relative to the estimated position of the moving fingertip. This more veridical perception of finger position when tactile stimuli are jointly presented with sounds can have implication for the design of efficient devices that need to be explored haptically.

## Supplementary Information

Below is the link to the electronic supplementary material.

Supplementary file1 (DOCX 2065 KB)

## Data Availability

Data for individual stimulus-response pairs is available from Zenodo (https://zenodo.org/records/11235911). Both experiments were registered on OSF: Experiment 1 (10.17605/OSF.IO/Y4GBN). Experiment 2 (10.17605/OSF.IO/X69UZ).

## Code availability

All experimental and analysis code used for this study is available from: https://github.com/Espositogiulia/psi_ATi_active_touch
