# Supplementary material for: Integration of auditory and tactile inputs to localize haptic stimuli during active touch
Source: Atten Percept Psychophys. 2026 Feb 23;88(3):74. doi: 10.3758/s13414-026-03223-w (PMC12929270; doi:10.3758/s13414-026-03223-w)
Supplement: Supplementary file 1 — Supplementary file1 (DOCX 2065 KB) [file 13414_2026_3223_MOESM1_ESM.docx]

**Supplementary materials**


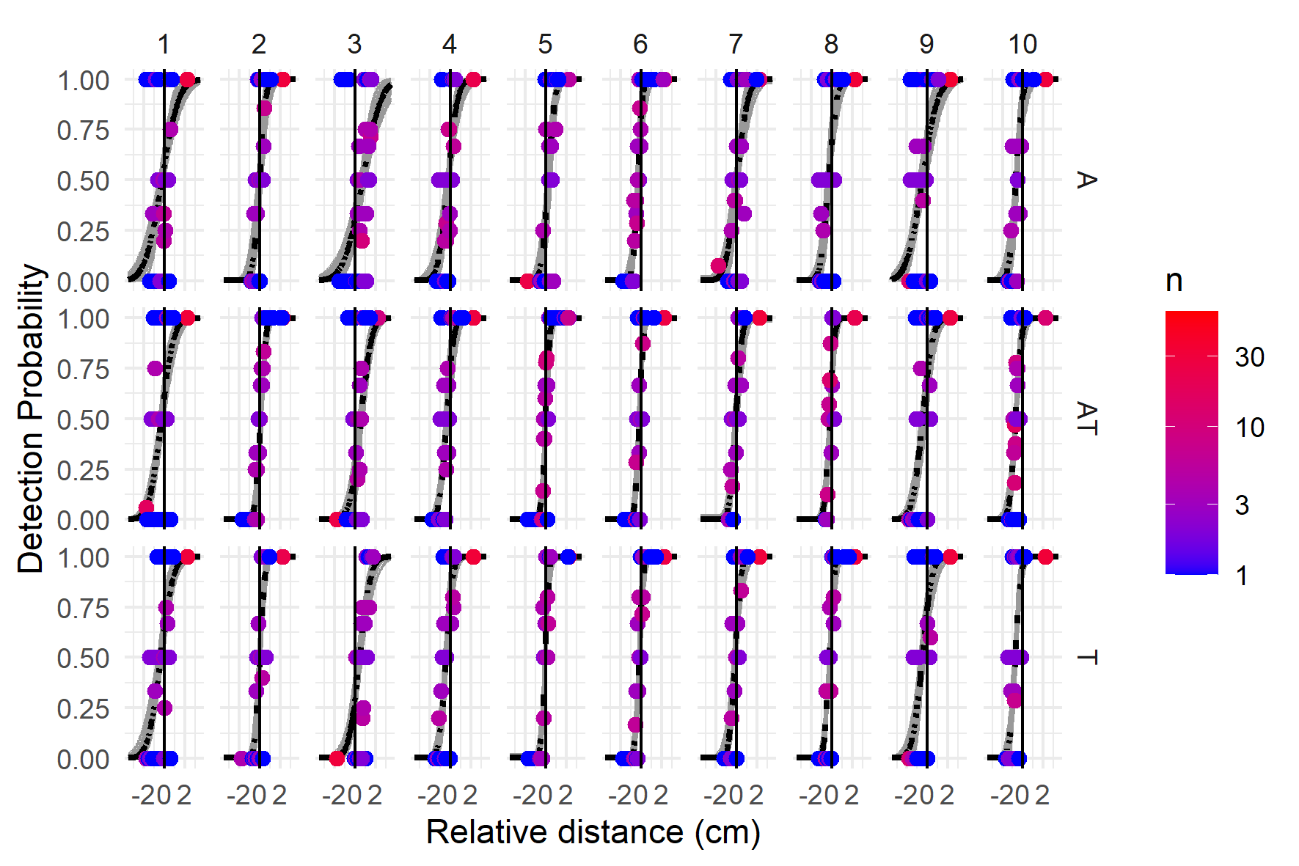


**Supplementary Figure 1. Individual participants’ fits of the model (experiment 1; Participants 1-10).** For each participant (columns) and condition (rows), the detection probabilities for the various delays are depicted as dots, color-coded based on the number of trials used to compute the plotted probability. The PF constructed using the most likely parameter (median) is plotted in black. 100 PFs constructed using random draws from the posterior distribution of the parameters are plotted in grey (uncertainty around the true parameter values).


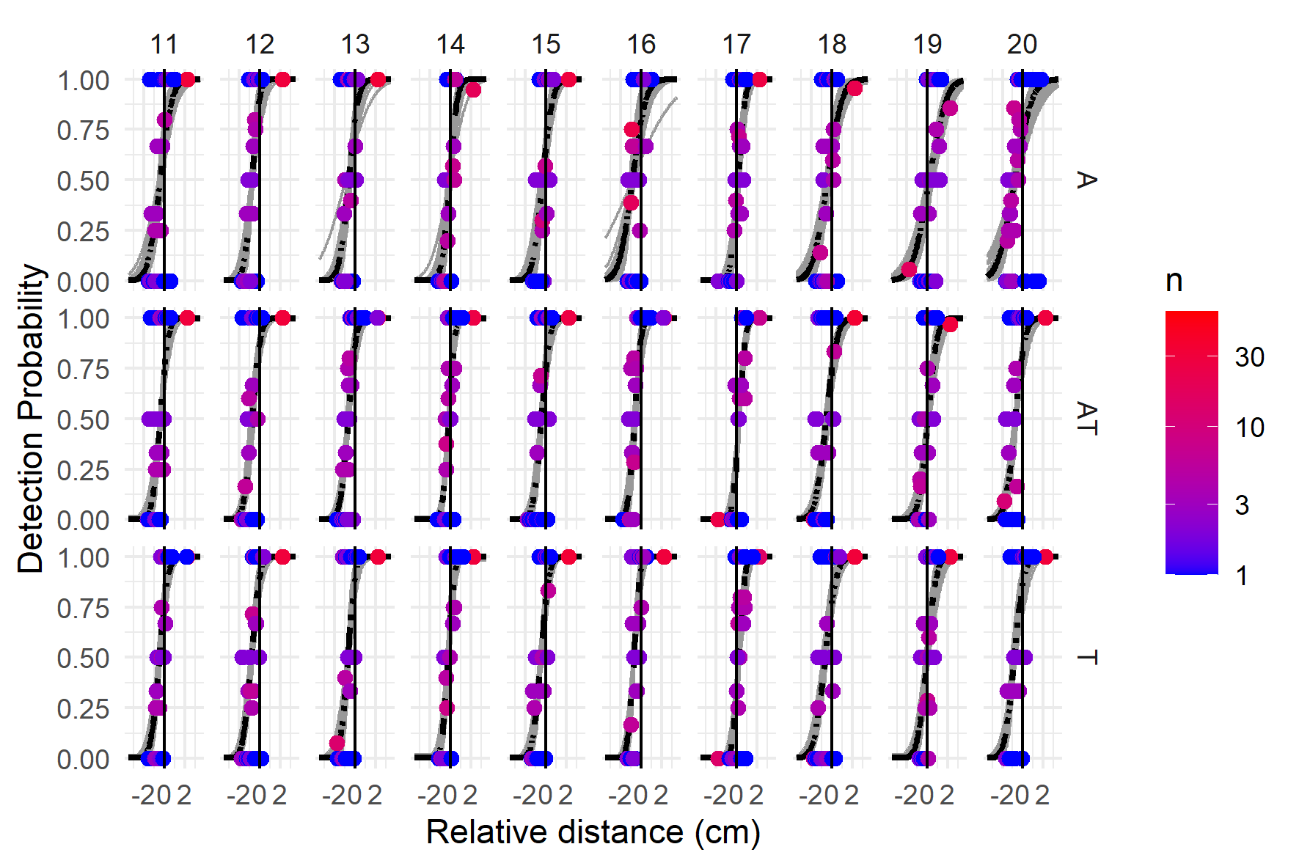


**Supplementary Figure 2. Individual participants’ fits of the model (experiment 1; Participants 11-20).** For each participant (columns) and condition (rows), the detection probabilities for the various delays are depicted as dots, color-coded based on the number of trials used to compute the plotted probability. The PF constructed using the most likely parameter (median) is plotted in black. 100 PFs constructed using random draws from the posterior distribution of the parameters are plotted in grey (uncertainty around the true parameter values).


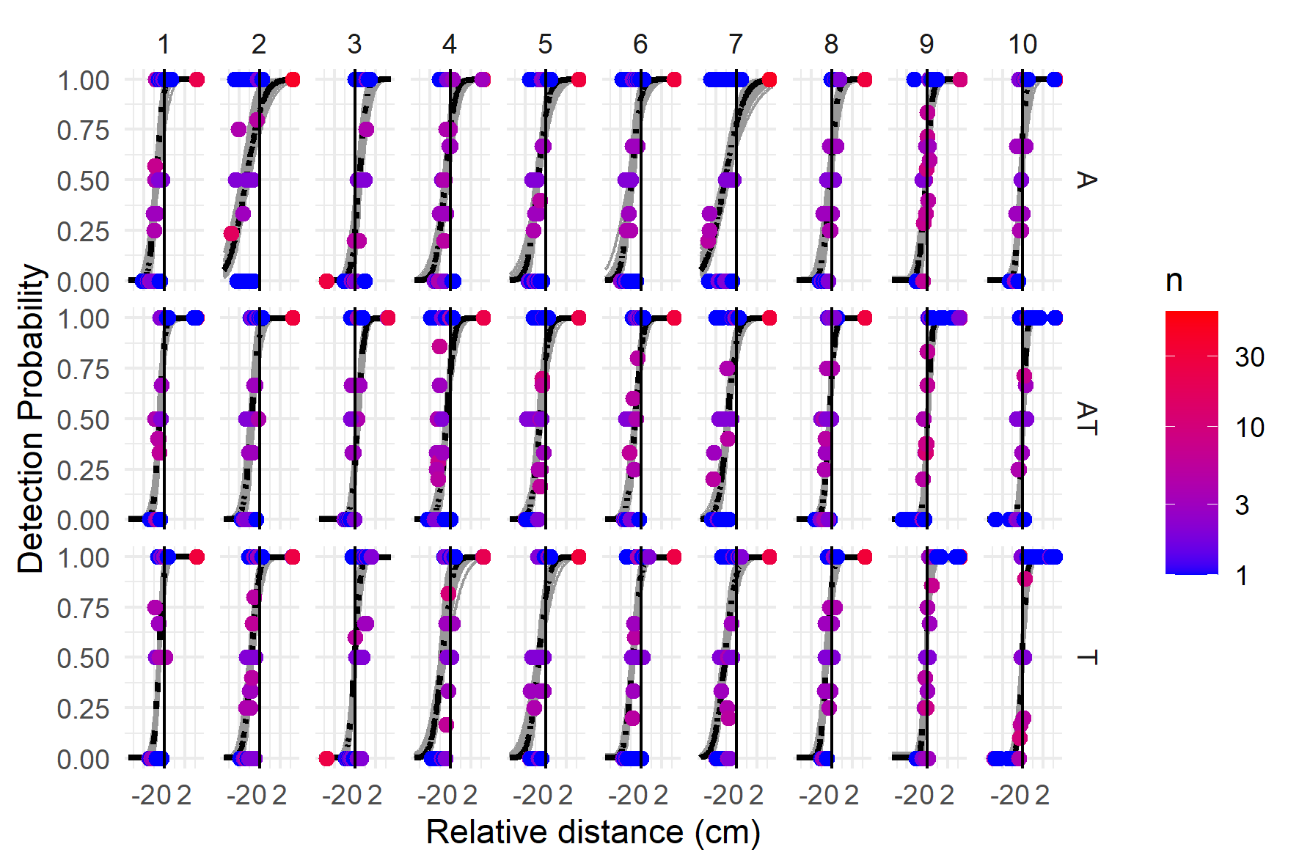


**Supplementary Figure 3. Individual participants’ fits of the model (experiment 2; Participants 1-10).** For each participant (columns) and condition (rows), the detection probabilities for the various delays are depicted as dots, color-coded based on the number of trials used to compute the plotted probability. The PF constructed using the most likely parameter (median) is plotted in black. 100 PFs constructed using random draws from the posterior distribution of the parameters are plotted in grey (uncertainty around the true parameter values).


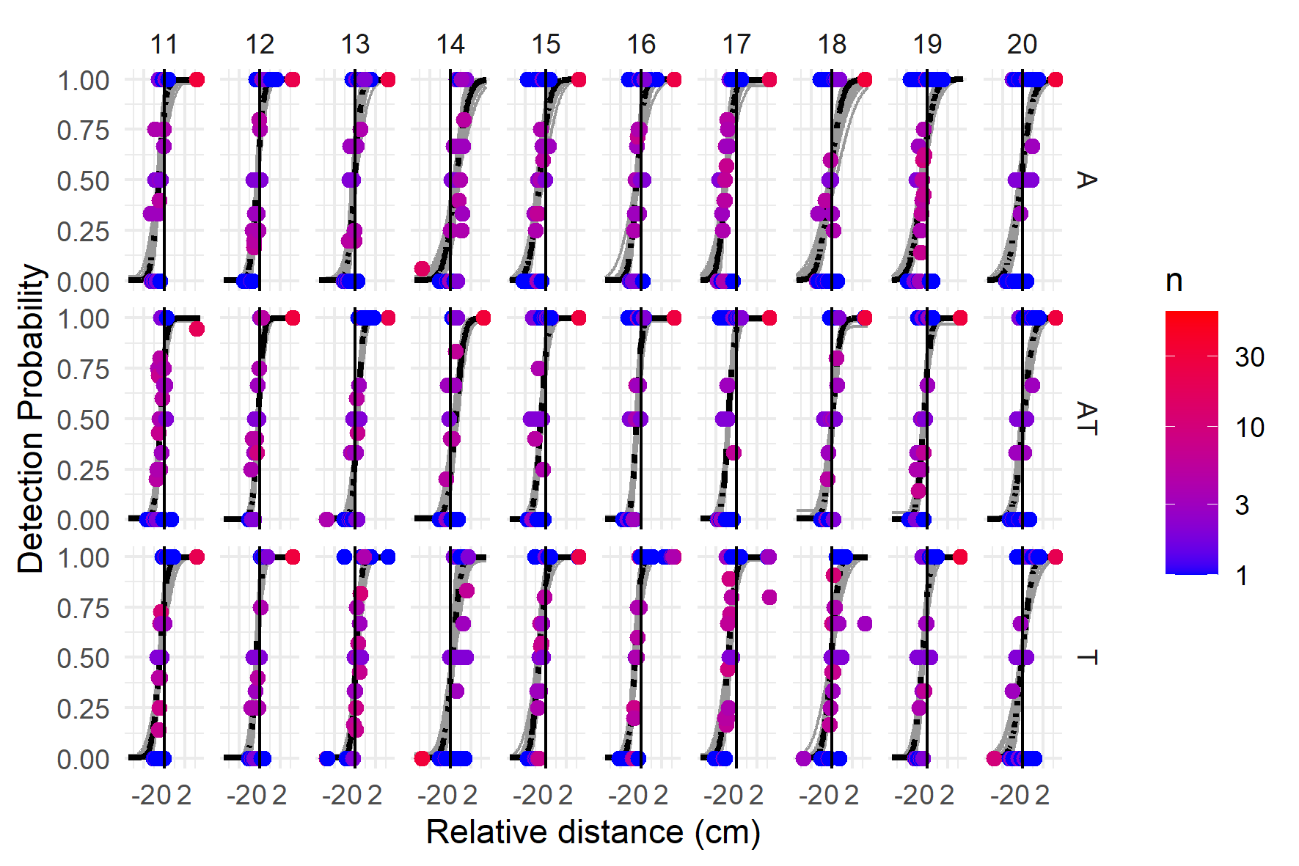


**Supplementary Figure 4. Individual participants’ fits of the model (experiment 2; Participants 11-20).** For each participant (columns) and condition (rows), the detection probabilities for the various delays are depicted as dots, color-coded based on the number of trials used to compute the plotted probability. The PF constructed using the most likely parameter (median) is plotted in black. 100 PFs constructed using random draws from the posterior distribution of the parameters are plotted in grey (uncertainty around the true parameter values).
